# Comprehensive dementia screening identifies patients with poor health outcomes and higher healthcare utilization

**DOI:** 10.1093/geroni/igaf122.4259

**Published:** 2025-12-31

**Authors:** Nabeel Qureshi, Pamela Roberts, Nancy Sicotte, Drew Hirsch, Mitzi Gonzales, Sarah Kremen, Zaldy Tan

**Affiliations:** Cedars-Sinai Medical Centers, Los Angeles, California, United States; Cedars-Sinai Medical Centers, Los Angeles, California, United States; Cedars-Sinai Medical Centers, Los Angeles, California, United States; Cedars-Sinai Medical Center, Los Angeles, California, United States; Cedars-Sinai Medical Center, Los Angeles, California, United States; Cedars-Sinai Medical Center, Los Angeles, California, United States; Cedars-Sinai Medical Center, Los Angeles, California, United States

## Abstract

There are 6.9 million persons living with dementia in the United States, and over 40% of hospitalized patients age 70+ have dementia. Detection rates in hospitals are less than 50%, likely underestimating the true burden. We implemented a comprehensive program to identify cognitive impairment at hospital admission, using a tripartite approach (EHR alerts for dementia, and administration of the 4AT and AD8) in inpatient medical/surgical units. Patients were grouped by screening results and delirium status. We conducted Poisson, logistic, and generalized linear regressions to assess differences in length of stay (LOS), readmission rates, prior hospitalizations, and costs between those with and without cognitive impairment. Over the first 16 months, we identified 11,180 patient encounters. Diagnosed dementia patients had lower LOS (7.5 vs. 8.3 days, p < 0.001), while those with undiagnosed cognitive impairment had higher LOS (9.6 vs. 8.3 days, p < 0.001). Diagnosed dementia patients had a higher probability of 90-day readmission (17.5% vs. 15.1%, p < 0.05), and more hospitalizations in the prior 6 months (6.5 vs. 4.5, p < 0.001). Patients with potential cognitive impairment but no delirium also had more prior hospitalizations (5.1 vs. 4.5, p < 0.01). Patients with any cognitive impairment incurred lower costs (502K vs. 573K, p < 0.001), with differences observed primarily among those discharged home. Implementing systematic screening for cognitive impairment at hospital admission reveals significant differences in health outcomes by dementia status. Early recognition and tailored care strategies for cognitively impaired patients are crucial to improving hospital outcomes.

